# Selective inhibition of PfA-M1, over PfA-M17, by an amino-benzosuberone derivative blocks malaria parasites development in vitro and in vivo

**DOI:** 10.1186/s12936-017-2032-4

**Published:** 2017-09-21

**Authors:** Lotfi Bounaadja, Marjorie Schmitt, Sébastien Albrecht, Elisabeth Mouray, Céline Tarnus, Isabelle Florent

**Affiliations:** 1 Molécules de Communication et Adaptation des Microorganismes, (MCAM, UMR7245), Muséum National Histoire Naturelle, Sorbonne Universités, CNRS, CP 52, 57 Rue Cuvier, 75005 Paris, France; 2 Laboratoire de Chimie Moléculaire, CNRS-UMR7509, Université de Strasbourg, 67037 Strasbourg Cedex 2, France; 3 Laboratoire de Chimie Organique et Bioorganique, EA4566, Université de Haute Alsace, 68093 Mulhouse Cedex, France

**Keywords:** Malaria, *Plasmodium falciparum*, M1 aminopeptidase, Chemotherapy, Amino-benzosuberone derivative

## Abstract

**Background:**

*Plasmodium falciparum* M1 family aminopeptidase is currently considered as a promising target for anti-malarial chemotherapy. Several series of inhibitors developed by various research groups display IC50/Ki values down to nM range on native PfA-M1 or recombinant forms and block the parasite development in culture at µM to sub-µM concentrations. A handful of these inhibitors has been tested on murine models of malaria and has shown anti plasmodial in vivo activity. However, most of these inhibitors do also target the other neutral malarial aminopeptidase, PfA-M17, often with lower Ki values, which questions the relative involvement and importance of each enzyme in the parasite biology.

**Results:**

An amino-benzosuberone derivative from a previously published collection of chemicals targeting specifically the M1-aminopeptidases has been identified; it is highly potent on PfA-M1 (Ki = 50 nM) and devoid of inhibitory activity on PfA-M17 (no inhibition up to 100 µM). This amino-benzosuberone derivative (T5) inhibits, in the µM range, the in vitro growth of two *P. falciparum* strains, 3D7 and FcB1, respectively chloroquino-sensitive and resistant. Evaluated in vivo, on the murine non-lethal model of malaria *Plasmodium chabaudi chabaudi,* this amino-benzosuberone derivative was able to reduce the parasite burden by 44 and 40% in a typical 4-day Peters assay at a daily dose of 12 and 24 mg/kg by intraperitoneal route of administration.

**Conclusions:**

The evaluation of a highly selective inhibitor of PfA-M1, over PfA-M17, active on *Plasmodium* parasites in vitro and in vivo, highlights the relevance of PfA-M1 in the biological development of the parasite as well as in the list of promising anti-malarial targets to be considered in combination with current or future anti-malarial drugs.

**Electronic supplementary material:**

The online version of this article (doi:10.1186/s12936-017-2032-4) contains supplementary material, which is available to authorized users.

## Background

Malaria is an infectious disease due to five protozoan species belonging to the *Plasmodium* genus, *Plasmodium falciparum* being responsible for the most severe lethal forms [1]. Currently, 214 million new malaria cases are recorded per year, resulting in approximately 438,000 deaths [2]. *Plasmodium* parasites are transmitted from human to human by the blood-feeding female *Anopheles* mosquitoes and undergo a complex life-cycle both in human and vector [3]. Although the development of anti-malarial drugs and vector control strategies have contributed to reduce the malaria burden during the last decade, notably through the usage of artemisinin-based combination therapy and insecticide-impregnated bed nets, half of the worldwide population is still exposed to malaria [1]. A tremendous threat remains since all commercially available anti-malarial drugs are facing parasite chemoresistance issues and no efficient vaccine is yet commercialized [1]. The need to further develop alternative or complementary anti-malarial strategies is, therefore, of high priority.

The identification of novel chemical classes of compounds (novel scaffolds) hitting new types of targets is necessary to propose other anti-malarial drugs potentially able to cope with the current chemoresistance status of malaria parasites [4, 5]. Such scaffolds emerge from a combination of “phenotypic” screenings where thousands of compounds are tested on parasite growth [6] and “target-oriented” screenings that are focusing on specific targets [7]. Among such targets are proteases, known to be involved in generic as well as specific metabolic pathways, such as the haemoglobin digestion cascade, that occurs within the parasite acidic food vacuole (FV) and contributes to provide most of the amino-acids necessary to the parasite metabolism, at least during its intra-erythrocytic life [8–10]. Indeed, despite having a limited capacity to synthetize amino acids de novo [11–13], the parasite has developed over evolution a complex pathway, involving a cascade of proteolytic enzymes from at least three classes (cysteine-, aspartic- and metallo-proteases), allowing the progressive digestion of ~ 75% of the haemoglobin of its host cell into free amino-acids [8, 12, 14–16]. Haemoglobin being poor in methionine, cysteine, glutamine and glutamate and containing no isoleucine, additional amino acids are exogenously imported through specific transporters notably isoleucine and methionine [17–19]. The various proteolytic enzymes contributing to the haemoglobin digestion and located within the FV have been extensively studied as potential targets of anti-malarials and belong to several classes of peptidases among which aspartic (plasmepsins), cysteine (falcipains) and metallo (falcilysin) endopeptidases, a dipeptidase and aminopeptidases [8, 9, 20]. Whether the free amino-acids are generated by these latter within the FV or at the level of the cytoplasm remains controversial [10, 20–24].

Among the nine aminopeptidases that are encoded in the *P. falciparum* genome [25], two are main contributors of this amino acids pool in the red blood cells asexual stage: PfA-M1 and PfA-M17. Both are encoded by single copy genes (PF3D7_1311800.1 for PfA-M1 and PF3D7_1446200.1 for PfA-M17, [26]). They have distinct active site architecture, belonging respectively to the M1 and M17 family of metallo proteases [27, 28]. Enzymatic studies using either native or recombinant forms of these enzymes have indicated that they also have a distinct, partially overlapped, substrate specificity suggesting non-redundant functions, by contrast to the endoproteases involved in the early steps of haemoglobin digestion (plasmepsins and falcipains) that are partly redundant [8, 29, 30]. PfA-M1 has the broadest N-terminal amino acids substrate specificity hydrolyzing preferably leucine, alanine, arginine, and phenylalanine, while PfA-M17 has much narrower specificity for leucine [19, 31–34]. Notably, each enzyme displays an optimal activity at neutral pH from 7.4 (PfA-M1) to 8 (PfA-M17) although PfA-M1 has been shown to be at least partially active at the FV acidic pH [21–23, 31].

PfA-M1, initially discovered through the isolation of its gene, was then affiliated to the highly conserved M1 family of metalloprotease with an active site closer to that of *Escherichia coli* PepN than to that of the mammalian members (including APN, APA, IRAP, ERAP2) [24]. The first biochemical and enzymatic studies were done using the purified native enzyme, revealing its optimal activity at pH 7.4, its dependence on zinc, and its complex maturation in iRBC, from a full length ~ 126 kDa form named p126 to three forms of ~ 120, ~ 96 and ~ 68 kDa named p120, p96 and p68 [21, 24, 35, 36]. The p35 C-terminal domain remains associated to the p68 form to yield the p68/p35 form of the enzyme [35], the in vivo meaning and/or relevance of this cleavage needing to be understood. A recombinant form of the enzyme corresponding to monomeric p96 was produced in *Escherichia coli* allowing the determination of its 3D structure in 2009, including, later a variant of PfA-M1 in which the S1 pocket was altered [31, 37]. Direct screening experiments on the native enzyme [38, 39] then on various recombinant forms as well as docking investigations [40, 41] have provided a series of inhibitors that display submicromolar to dozen nanomolar Ki/IC50 values and submicromolar IC_50_ values on parasites growth [10, 20, 31, 36, 38, 39, 42–45]. Some inhibitors have also been shown to decrease or suppress parasitaemia in murine models of malaria [46, 47].

It is nevertheless important to underline that most of these PfA-M1 inhibitors do also target the hexameric enzyme PfA-M17 [32, 34]. Inhibitors of PfA-M17 were discovered through various screening programs [45, 48] and most of them do inhibit both PfA-M1 and PfA-M17 with sometimes highly distinct Ki values [45]. For example, bestatin, the reference inhibitor of zinc-aminopeptidases is reported to be more potent on PfA-M17 (Ki 0.025–0.6 µM) than on PfA-M1 (0.2–1.5 µM) [10, 20, 31, 48, 49]. So far, very few specific inhibitors for either PfA-M17 or PfA-M1 have been identified with the notable exceptions reported by Harbut et al. who described one of each [43]. Unfortunately, these enzyme-specific inhibitors were not evaluated in vivo and, therefore, the in vivo relevance of each of these two metallo-aminopeptidases remains to be specifically investigated. Indeed, the few M1/M17 inhibitors that have been studied in vivo do target both enzymes and at first the M17 metalloenzyme (Table 1).

**Table 1:** Summary of in vitro and in vivo PfA-M1 inhibitors properties

	Structure	Ki rPfA M1 (nM)	Ki rPfA M17 (nM)	IC_50_ in vitro (µM)	In vitro strain	In vivo model	In vivo parasitaemia reduction (%)	References
CHR-2863		1413.41 ± 123.74	75.96 ± 22.86	0.37	3D7	*P. c. chabaudi*/C57/BL6j	43% (25 mg/kg)	[46]
				0.376	K1			
							52% (50 mg/kg)	
							66% (100–50 mg/kg)	
Bestatin		1673.00 ± 238.05	613 ± 201.33	5	3D7	*P. c. chabaudi*/8 weeks old C57/BL6j	34% at 100 mg/kg, twice a day	[46, 47]
				12–21	Dd2			
			25 ± 1.2	11–15	D10			
				8–14	3D7			
Malarone		NA	NA	–	–	*P. c. chabaudi*/C57/BL6j	100% at 14 mg/kg	[46]
Chloroquine		–	–	10.1 ± 3.8	3D7	*P. c. chabaudi*/8 weeks old C57BL/6j	85% at 5 mg/kg	[47]
				138.0 ± 21.1	FcB1			
Compound 4		79	13.2 ± 0.5	20–40	Dd2	*P. c. chabaudi*/8 weeks old C57BL/6j	92% at 100 mg/kg, twice a day	[31, 47]
				46–75	D10			
				24–62	3D7			
T5		50	> 100 µM	11.2 ± 3.4	3D7	*P. c. chabaudi*/BALB/c	44 and 40% at 12 and 24 mg/kg/day	*this work*
				6.5 ± 2.4	FcB1			

*NA* data not available

The current report relates the in vitro and in vivo inhibitory activity of a novel highly specific inhibitor of PfA-M1, over PfA-M17, mentioned as T5. This compound was sorted out from a collection of previously described M1-family inhibitors based on a benzosuberone (7-amino-benzocycloheptan-6-one) chemical scaffold [50, 51]. Its in vitro and in vivo evaluation will contribute to a better understanding of the functional involvement of PfA-M1 in the parasite metabolism and its potentiality as an appropriate anti-malarial target.

## Methods

### Production of recombinant enzymes

Synthetic genes (Genecust, Luxembourg) encoding residues 192–1085 of native *P. falciparum* alanyl aminopeptidase PfA-M1 (PlasmoDB PF3D7_1311800) or to residues 84–605 of native *P. falciparum* leucyl aminopeptidase PfA-M17 (PlasmoDB PF3D7_1446200), were cloned into the pET45b (Novagen) vector, (into *KnpI* and *SalI* sites for PfA-M1 and *BamHI* and *SalI* sites for PfA-M17) which appended a N-terminal hexahistidine tag. *Escherichia coli* Rosetta2 (DE3) bacteria (Novagen) were transformed with the plasmids after their validation by Sanger sequencing (Beckman Coulter Genomics). Bacterial cultures were grown in auto-induced LB medium (Merck) supplemented with appropriate antibiotics (carbenicillin 50 µg/mL, chloramphenicol 34 µg/mL) during 24 h at 25 °C prior to bacterial extract preparations with BugBuster^TM^ (Novagen). The clarified lysates were loaded onto Ni_2_-charged HisTrap column (GE Healthcare) equilibrated in phosphate buffer supplemented with 20 mM imidazole, washed in the same buffer, and bound recombinant proteins were eluted in 80 mM imidazole for PfA-M1 or 200 mM imidazole for PfA-M17, in phosphate buffer. Eluted fractions were then purified by size exclusion chromatography on a Superdex 200 10 300 (equilibrated with Tris HCl 50 mM, NaCl 200 mM, ZnCl_2_ 10 µM, pH 7.4 for PfA-M1 and with Hepes 50 mM, NaCl 300 mM, ZnCl_2_ 10 µM, 5% glycerol, pH 8.5 for PfA-M17) using an Äkta purifier chromatography system.

### Enzyme assays and kinetic analysis

Enzyme activities of rPfA-M1 and rPfA-M17, at 10 and 60 nM respectively, were determined by measuring continuously the release of para-nitroaniline at 405 nm with alanine para-nitroanilide as substrate for rPfA-M1 (KM = 1.5 mM), and leucine para-nitroanilide for rPfA-M17 (KM = 0.5 mM). Assays were carried out on a HP/Agilent UV–Visible diode array spectrophotometer 8453, at 30 °C, in Tris HCl 50 mM, pH 7.4 for rPfA-M1, and pH 8 for rPfA-M17 with a final DMSO concentration of 1%. Initial velocities were measured with increasing concentrations of inhibitors and Ki values were determined by Dixon plots [52].

### Tested compounds

The inhibitor T5 has been synthesized as previously described [50, 51] and a 20 mM stock solution was prepared in DMSO. Chloroquine and bestatin were purchased in Sigma-Aldrich and were resuspended in sterile water (1 mM) and in DMSO (20 mM) as stock solutions, respectively. Pharmacokinetic properties are detailed in the supplementary data section.

### Cytotoxicity studies

Cellular cytotoxicity was evaluated using Rat skeletal muscle cell line L6, maintained at 37 °C in RPMI 1640 medium supplemented with 10% (v/v) fetal calf serum. Cells were seeded in 96-well-plates (20,000 cells/mL) and incubated for 24 h, then, L6 cells were treated with the drug for 72 h. After incubation, the cell growth medium was replaced by 100 μL RPMI 1640 containing 20% (v/v) Alamar Blue (Thermofisher, France). Fluorescent viable cells were monitored after 5 h of incubation at 37 °C, at a wavelength of 530 nm for excitation and 590 nm for emission, in a FL600 luminescence spectrometer (Biotek, France). CC_50_, corresponding to drug concentrations causing 50% L6 cell proliferation inhibition, were calculated from the drug concentration—response curves.

### In vitro studies against *Plasmodium falciparum*

In vitro antiplasmodial assays were performed on the *P. falciparum* chloroquine-resistant FcB1 (Columbia) and chloroquine-susceptible 3D7 (Tanzania) strains as previously described [38, 39]. Briefly, asynchronous parasites were cultured in human erythrocytes at 37 °C in RPMI 1640 medium (Gibco-BRL) supplemented by 10% (v/v) heat-inactivated (at 56 °C for 45 min) human serum (containing 25 mM HEPES adjusted at pH 7.5, 27.5 mM NaHCO_3_, 11 mM glucose, 100 UI/mL Penicillin and 100 µg/mL Streptomycin) under an atmosphere of 3% CO_2_, 6% O_2_, and 91% N_2_, as described by Trager and Jensen [53]. An asynchronous parasite culture (1% parasitaemia and 1% final haematocrit) was added to the wells of 96-well plates with serial dilutions of drugs prior to a 24 h incubation at 37 °C in a 5% CO_2_ incubator, with 200 μL total volume per well. After incubation, 0.5 µCi of ^3^H-hypoxanthin (11.1 mCi/mmol; Perkin-Elmer, France) was added per well and plates were returned to 37 °C in 5% CO_2_ incubator for a further incubation of 24 h. On the following day, test plates were frozen at −20 °C. Thawed cell lysates have been counted onto glass-fiber filters in a liquid scintillation spectrometer. The radioactivity incorporated into the cultures allowed the calculation of the percentage of growth inhibition by comparison to control cultures without inhibitor (but with equivalent amount of solvent). The concentration of chemical resulting in 50% of parasite growth inhibition (IC_50_) was determined by non-linear regression analysis of the data using GraphPad Prism 6 (GraphPad Software, CA). Results were expressed as the mean values ± standard deviations determined from independent experiments. The DMSO concentration in the assays never exceeded 0.5% and bestatin and chloroquine (Sigma-Aldrich) were used as standard controls. All compounds were diluted in RPMI 1640 medium supplemented by 10% (v/v) heat-inactivated human serum for in vitro assays.

### Morphological alterations analysis

FcB1 parasites, cultured in vitro as described above, were first enriched for late stages using Plasmion [54] and were then seeded to fresh red blood stages, followed, after 6 h of culture, by 5% sorbitol treatment [55] to yield a synchronized culture of early stages parasites (0–6 h post invasion). The morphological effects due to T5 treatments were then assessed throughout the parasite life cycle, using 96-well plates seeded with these synchronized parasites supplemented with either T5 (at three concentrations), corresponding solvent (untreated control), or control inhibitor (E64 at 10 µM). The parasite morphology was done by Giemsa-stained blood smears examination at 12, 24, 36 and 48 h post invasion. Concentrations used for T5 were 4, 20 and 40 µM.

### Sequence alignment and PcA-M1 3D modeling

In silico analyses of M1 protein sequences from several *Plasmodium* parasites were performed using the ClustalW program of MEGA version 6 [56]. Aminopeptidase M1 protein sequences from *P. falciparum* (O96935), *Plasmodium c. chabaudi* (XP_745040.2), *Plasmodium berghei* (XP_680130.1) and *Plasmodium yoelii* (XP_729336.1) were obtained from EMBL databases. Protein structure homology modeling of the PcA-M1, PbA-M1 and PyA-M1 were based on the template structure of PfA-M1 (PDB code: 4J3B) [37] and was performed by the automated protein structure homology-modelling server SWISS-MODEL [57].

### In vivo studies against *Plasmodium chabaudi chabaudi*

The in vivo anti-malarial activity of T5 was evaluated using the non-lethal murine *P. c. chabaudi* 864VD strain [58] in 9-weeks old BALB/c female mice (Janvier, France), using bestatin as reference and chloroquine as positive control. Animals were acclimatized for three weeks to the experimental environment. All molecules were evaluated using an adapted suppressive Peters 4-day test [59].

Briefly, tested molecules were suspended daily in vehicle consisting of a DMSO/5% glucose solution: 10/90. Five groups of six mice were used. Group 1: vehicle control; group 2: T5 at the dose of 12 mg/kg in vehicle; group 3: T5 at the dose of 24 mg/kg in vehicle; group 4: bestatin at the dose of 100 mg/kg in vehicle; group 5: chloroquine at the dose of 5 mg/kg in PBS. Mice (average weight 20 g) from all five groups, housed under regular daylight, were infected on day 0 at 9.30 am, intraperitoneally, with 10^6^ red blood cells infected with *P. c. chabaudi* 864VD strain. The five groups of six mice were injected daily from day 0 to day 3 at 11.30 am with the corresponding molecules freshly prepared in vehicle or PBS. Kinetics of infection were then followed daily for 25 days, by microscopic inspection of Giemsa-stained blood tail smears performed daily at ~ 10 a.m. (ring stages). Mice were housed in the animal care facility of the UMR7245 CNRS-MNHN laboratory under specific-pathogen-free conditions.

### Statistical analysis

Percentages of parasitaemia were measured for all animals in each treatment group. Peaks of parasitaemia were expressed as the mean ± standard error of the mean, for each condition, and comparison of treatment groups to the vehicle group was conducted using the two-tailed Mann–Whitney U test. Representation and data analyses were performed with the Graph Pad Prism6 software. Only p < 0.05 was considered statistically significant.

## Results

### Selective inhibition of PfA-M1, over PfA-M17, by a benzosuberone derivative

**Fig. 1:**
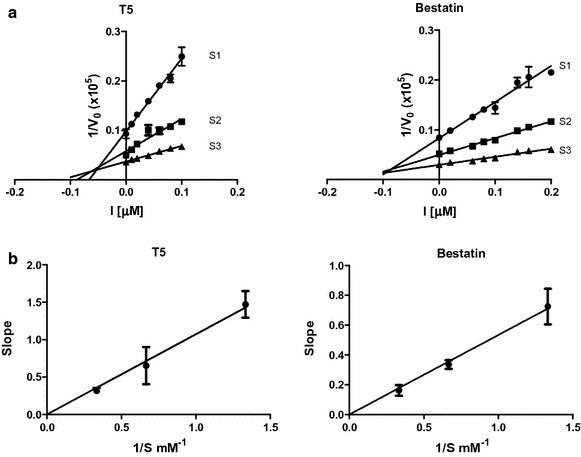
Kinetic analysis of PfA-M1 inhibition by compound T5. **a** PfA-M1 activity was measured with increasing Ala-pNA chromogenic substrate concentrations (S1 = Km/2, S2 = Km and S3 = 2 km) over a range of inhibitor concentrations [I], from 0.01 to 0.1 μM. Dixon linearization plots are shown for PfA-M1 enzyme versus T5 or bestatin. **b** Competitive inhibition was assessed by plotting the slope of the primary Dixon plot versus the inverse of the substrate concentration and was inferred by the straight line passing through the origin. Results are presented as the mean ± SEM from three independent measurements

The inhibitory effect of T5 was investigated on the enzymatic activity of rPfA-M1 and rPfA-M17 to test its selectivity. The Ki value determined for T5 against rPfA-M1 was 50 nM, with no significant inhibition of rPfA-M17 up to 100 μM. Under the experimental conditions used in this current study, bestatin displayed quite close inhibition constants on both enzymes with Ki values of 100 nM on rPfA-M1 and 400 nM on rPfA-M17, confirming its lack of selectivity. Dixon plots and slope replots indicated competitive inhibition of T5 against PfA-M1 (Fig. 1a, b). These experiments ascertain the selectivity profile of the evaluated compound, with high affinity towards PfA-M1 and a selectivity index superior to 2000.

### High sequence and structural conservation between PfA-M1 and its rodent parasites orthologues

**Fig. 2:**
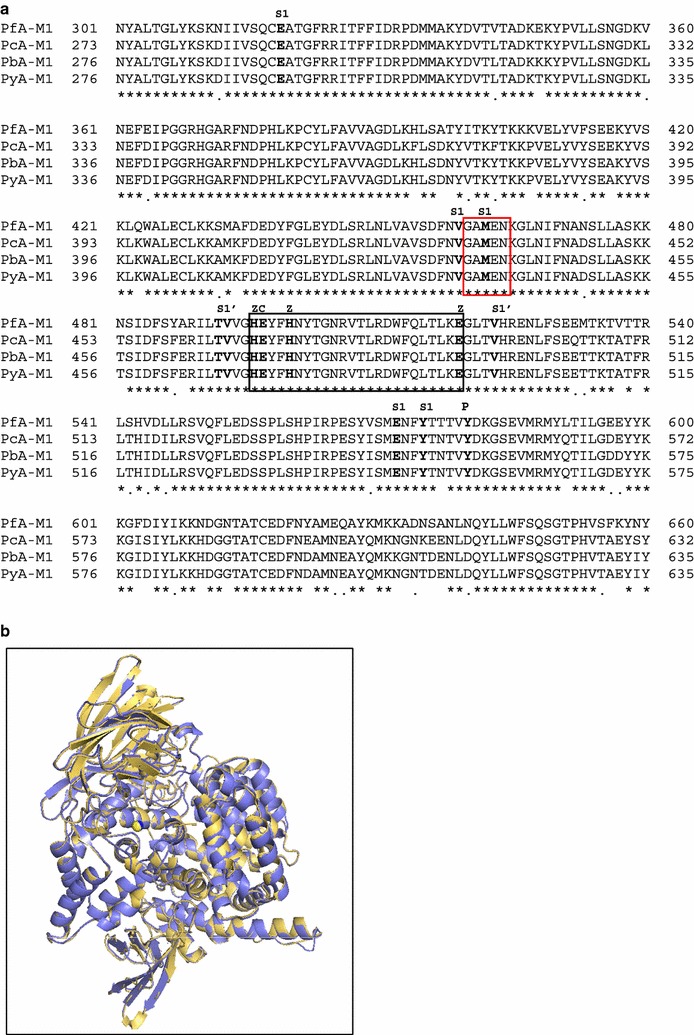
Comparison of M1 aminopeptidases from *P. falciparum*, *P. c. chabaudi*, *P. berghei* and *P. yoelii*. **a** Aminopeptidase M1 sequences from *P. falciparum* (O96935, PfA-M1), *P. c. chabaudi* (XP_745040.2, PcA-M1), *P. berghei* (XP_680130.1, PbA-M1) and *P. yoelii* (XP_729336.1, PyA-M1) were used for the multi alignment corresponding to domain 301–660 of the native PfA-M1 sequence. The active site HExxHx_18_E motif and the conserved GAMEN sequence are boxed in black and red, respectively. Identical (*) and conserved (.) amino acids between the four sequences are indicated. Key amino acids involved in S1 and S1′ subsites, in the ligation to zinc ion (Z), required for catalytic activity (C) and acting as putative proton donor (P) are indicated above alignment. **b** The 3D molecular model of PcA-M1 (blue) was compared to the 3D structure of PfA-M1 (PDB: 4J3B, yellow). Structures superposition was prepared using the PyMOL molecular graphics system

Murine models of malaria are currently used to test anti-malarial drug candidate in vivo. In this study, a non-lethal *P. c. chabaudi* model was selected because of similarities with *P. falciparum* in invasion of mature erythrocytes, synchronicity of erythrocytic cycle in vivo, antigenic variation and mechanism of resistance [60–62]. Interestingly, this murine malaria model has also been selected by the few groups that have so far tested PfA-M1/PfA-M17 inhibitors in vivo as indicated in Table 1 [46, 47]. Prior to undertaking these in vivo studies, the conservation at the sequence and structural levels of the rodent parasite orthologues of PfA-M1 was evaluated. The M1-aminopeptidase gene sequences were retrieved from EMBL databases for *P. c. chabaudi* (ENA accession number CDR16420.1), *P. yoelii* (ENA accession number EAA20901.1) and *P. berghei* (ENA accession number CAH98191.1). In silico analysis confirmed that each of these genes is present as a single copy in their respective genomes (information accessible at the reference Plasmo DB database [26]). The encoded amino acids sequences were then used for multiple alignments and 3D structure predictions, namely: *P. falciparum* (O96935), *P. c. chabaudi* (XP_745040.2), *P. berghei* (XP_680130.1) and *P. yoelii* (XP_729336.1). This alignment, centered on the active site domain [corresponding to positions 301–660 of the PfA-M1 native sequence (O96935)], is presented on Fig. 2a. It confirms the conservation of the key amino-acids involved in the catalytic activity such as the catalytic glutamyl residue (E497, PfA-M1 numbering) and the conserved zinc ion ligands (H496, H500 and E519, PfA-M1 numbering) in the HExxHx18E canonical sequence (residues 496–500, PfA-M1 numbering), as well as the proton donor (Y580) of MA-Clan/M1-family enzymes and the conserved GAMEN motif involved in substrate primary amine recognition [24, 63].

A 3D- model for PcA-M1 was generated based on the 3D-structure of PfA-M1 (PDB: 4J3B) to assess the structural identity between the two enzymes [37]. A superposition of PcA-M1 3D-modelled structure with that of rPfA-M1 confirmed their closely conserved active site architectures. The 3D-modelled structures of PbA-M1 and PyA-M1, built using the same approach, also revealed the highly conserved structures of the active sites for these M1-family malarial aminopeptidases. The choice of the non-lethal murine model *P. c. chabaudi,* therefore, appeared suitable to evaluate the in vivo effect of T5.

### In vitro and in vivo anti-malarial activity of T5

The ability of the benzosuberone derivative T5 to inhibit *P. falciparum* growth was examined in parasite cultures. The 50% inhibitory concentrations (IC_50_) of T5 against asynchronous 3D7 and FcB1 strains were 11.2 ± 3.4 and 6.5 ± 2.4 µM, respectively (Table 1), which is similar to bestatin (8.2 ± 1.9 µM for 3D7 and 9.0 ± 2.1 µM for FcB1). The cytotoxicity of the T5 compound was evaluated on L6 cells. The CC_50_ value obtained for T5 (CC_50_ = 141.0 ± 12.1 µM) allowed to calculate the selectivity index (SI) corresponding to the ratio of cytotoxicity and anti-malarial activity for both strains. T5 revealed a SI of 12.5 and 21.7 for 3D7 and FcB1, respectively.

As the inhibitory effect of T5 on *P. falciparum* was confirmed in vitro, T5 was further evaluated in a detailed in vitro ADME screen (see Additional file 1 for more details). In vivo pharmacokinetics (PK) studies showed rapid absorption after oral or intraperitoneal administration (3 mg/kg) but with limited exposure, moderate terminal half-life and large volume of distribution.

**Fig. 3:**
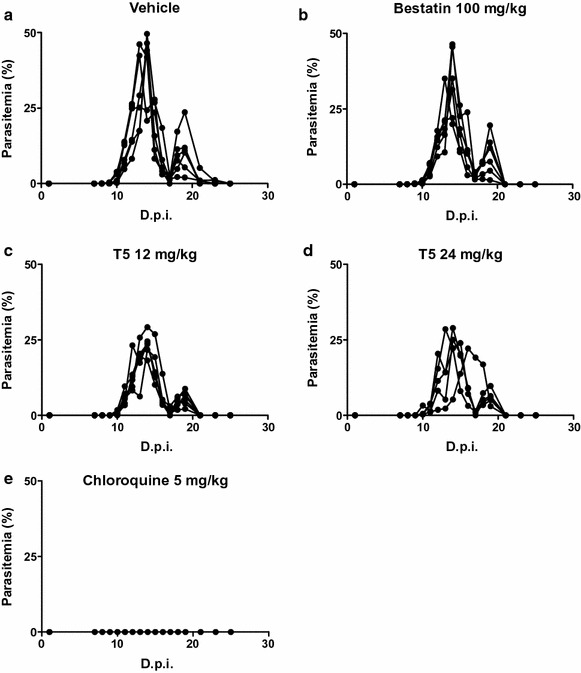
In vivo efficacy of T5, bestatin and chloroquine against *P. c. chabaudi* infection in mice. Female BALB/c mice infected with *P. c. chabaudi* 864VD at day 0 were treated daily for 4 days (0–3) with: vehicle only (**a**), 100 mg/kg of bestatin (**b**), 12 mg/kg of T5 (**c**), 24 mg/kg of T5 (**d**) or 5 mg/kg chloroquine (**e**). D.p.i. is days post-infection, n = 6 mice per group

Subsequently, the ability of T5 to reduce parasite proliferation in vivo was evaluated according to the 4-day suppressive test of Peters [59] using the non-lethal parasite model *P. c. chabaudi*. Four daily injections were performed, followed by a daily monitoring of parasitaemia by microscopic analysis until day 25 (Fig. 3).

**Fig. 4:**
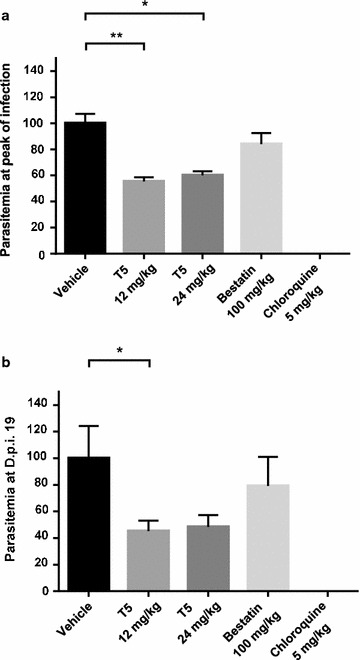
Comparative efficacy of anti-malarial in vivo treatments. **a** Parasitaemia at main infection peak was normalized on non-treated mice group (vehicle) to allow comparative evaluation of the respective daily treatments with: 12 mg/kg of T5, 24 mg/kg of T5, 100 mg/kg of bestatin and 5 mg/kg of chloroquine. **b** Parasitaemia at the recrudescence peak on day 19 post infection was similarly normalized and compared for the five mice groups. For each condition, data were analyzed using the Mann–Whitney U test for statistical significance. *p < 0.05; **p < 0.01

In non-treated infected mice, parasites were detected with a first peak of parasitaemia from 27.9 to 49.6% between day 13 and day 14 post-infection (Fig. 3a). After a transient recrudescence of the parasitaemia at day 19 post-infection, parasites could not be observed anymore. In infected mice treated with bestatin, a peak of parasitaemia from 22.0 to 45.5% was observed between day 13 and day 14 post-infection, and a second recrudescent peak was also observed at day 19 post-infection (Fig. 3b). In infected mice treated with T5, depending on the tested concentration, the peaks of parasitaemia spread from day 13 to day 16 post infection and presented values ranging from 19.6 to 29.2% and 22.2 to 37.3% for 12 and 24 mg/kg daily doses, respectively (Fig. 3c, d). In positive control infected mice, treated with the standard anti-malarial chloroquine at the daily dose of 5 mg/kg, the parasitaemia was below 0.02% at any time, corresponding to a complete elimination of the parasites (Fig. 3e). Figure 4a summarizes the efficacy of the various treatments by plotting the mean parasitaemia at the peak, for each group of treated mice, normalized by the parasitaemia at the peak for the control group (untreated infected mice). Such representation allows to visualize that 4 days treatment at a daily dose of 100 mg/kg of bestatin did not reduce significantly the parasite infection with a mean parasitaemia at 84% compared to the 100% for the control group, corresponding only to a 16% reduction of the parasitaemia, while a 4 days treatment at a daily dose of 12 or 24 mg/kg of T5 reduced significantly the infection by respectively 44.4 and 39.8%. A comparison of the mean parasitaemia reached by each group of mice for the recrudescence peak at day 19 was also represented (Fig. 4b), indicating that, similarly, the parasite reduction was no significant with bestatin (reduction of only 20.7%) while T5 could reduce the parasite burden by half (54.7% for 12 mg/kg and 51.5% for 24 mg/kg) compared to the vehicle.

Thus, T5 reduced the overall parasitaemia at the peak of infection and the recrudescence by 40–44%, at the both doses tested. Treatments with T5 at doses 12 and 24 mg/kg presented no significant differences in the parasiticidal activity that may suggest a potential limitation due the solubility and bioavailability issues for this compound.

### Parasite morphological alterations caused by T5 treatments

**Fig. 5:**
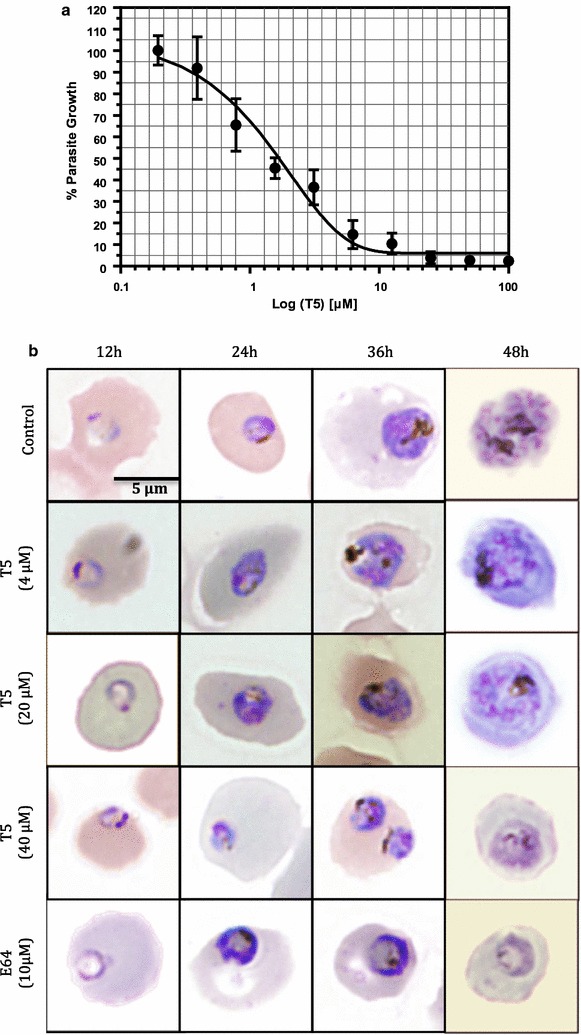
Effect of T5 on parasite morphology in vitro. **a** Dose–response curve for T5 on synchronized *P. falciparum* parasites (FcB1 strain, 0–6 h post invasion, 48-h application) allowing IC_50_ determination on synchronized parasites. **b** Morphological alterations following T5 applications at various concentrations: 4, 20 and 40 µM, compared to an untreated control and E-64 (10 μM) treated parasites. Drug treatments were carried out on synchronized cultures (0–6 h post invasion) and morphological evaluations were done: 12, 24, 36 and 48 h after

In order to evaluate the morphological alterations caused by T5 treatments, *P. falciparum* FcB1 parasites were synchronized (0–6 h post invasion) then exposed to three T5 doses corresponding to 2, 10 and 20 times the T5 IC_50_ (Fig. 5a), and their morphology was monitored at four different time points post invasion (p.i.): 12, 24, 36 and 48 h. Parasites treated with the three doses of T5 (Fig. 5b) exhibited a moderate swelling of the digestive vacuole at the trophozoite stage (36 h p.i.). At 48 h p.i. corresponding in controls to late schizont stages, a slowdown in the parasite development was observed for parasites treated with the highest dose of T5 (40 µM) that seemed to be blocked since no schizonts were observed at 48 h p.i. (Fig. 5b). In control slides treated with the cysteine protease inhibitor E64 (10 µM), a swelling of the digestive vacuole was also observed and this up to 48 h p.i. (Fig. 5b).

### Discussion and conclusion

Very few molecules selectively inhibiting PfA-M1 without inhibiting PfA-M17 have been so far described in the literature [43]. Obtaining such selectivity remains challenging since both aminopeptidases display a catalytic mechanism involving essential Zinc ions. Accordingly, most evaluated PfA-M1 inhibitors also target PfA-M17 and are generally more potent on PfA-M17 than on PfA-M1. A small number of inhibitors of PfA-M1/PfA-M17 has been tested in vivo and all these inhibitors are thus far documented to be more potent on PfA-M17 than on PfA-M1 (Table 1). Therefore, the chemotherapeutic relevance of PfA-M1 remains unrevealed at this time, due to dual non-selective inhibition on both targets.

According to current literature, malarial M1/M17 inhibitors present interesting in vitro potency against *P. falciparum*, although in vivo data remains scarce. The compound targeting malarial M1/M17 enzymes having the best curative effect to fight a *P. c. chabaudi* infection in vivo (92% inhibition) is the dual inhibitor phosphinic compound Co4 (compound 4) that targets both PfA-M1 (Ki 79 nM) and PfA-M17 (Ki 13.2 nM). Nevertheless, it should be noted that this compound has been tested in vivo following a different protocol, namely using 100 mg/kg doses, twice a day for 7 days, which corresponds to daily doses sixteen times higher than those used here with T5, and to a much longer treatment timing, close to doubled (7 days versus 4 days in the current study) [47]. Another example in the same murine malaria model concerns CHR-2863 (20 times more potent on PfA-M17 than on PfA-M1) with an interesting in vivo anti-malarial activity (by the Peters 4-day suppressive test) with 43 and 52% of inhibition at the doses of 25 and 50 mg/kg/day, respectively, and a 66% of inhibition when a 2 days treatment at 50 mg/kg/day was followed by a 2 days treatment with a dose of 100 mg/kg/day [46].

In the present work, T5, an amino-benzosuberone derivative, was identified as a very potent and selective inhibitor of PfA-M1 (Ki = 50 nM) versus PfA-M17 (Ki ≫ 100 µM). This compound was active against two *P. falciparum* strains grown in vitro, displaying IC_50_ of 6.5 ± 2.4 µM (FcB1) and 11.2 ± 3.4 µM (3D7) and showing a significant antiparasitic effect in vivo. Note that there are several orders of magnitude discrepancies between the Ki value of T5 on PfA-M1 and the IC50 value on parasite growth in vitro. Such a situation is currently encountered in the malaria field (see also compound 4 in Table 1) and is due to the fact that the inhibitors must cross several biological membranes to reach their targets. Indeed, similar discrepancies have been reported by others for PfA-M1 inhibitors [31, 42–44]. This PfA-M1 specific inhibitor was able to reduce the parasitaemia by ~ 44% in a non-lethal murine model of malaria, with only a daily dose of 12 mg/kg, in four daily intraperitoneal injections Peter’s protocol. Although this result seems modest, this compound T5 represents a new promising scaffold by targeting a malarial enzyme important for the parasite metabolism, an essential result in the current context of *P. falciparum* drug resistance. In addition, this new compound was more effective than a 100 mg/kg daily dose of bestatin during 4 days, a standard but non-selective inhibitor towards M1/M17 aminopeptidases.

The morphological alterations induced by T5-treatments, notably in both trophozoite and schizont stage parasites confirm a key role of PfA-M1 during the breakdown of haemoglobin but indicate a possible additional role in late stages. Importantly, T5 had no effect on the morphology of ring stage parasites. The digestive vacuole swelling observed at the three tested T5 concentrations, at 36 h post-invasion, was however lower than what was observed by Harbut and collaborators using the PfA-M1-inhibitor BTA on the 3D7 strain [43], which is in agreement with the studies previously carried out in this laboratory that PfA-M1 would be only marginally delivered to the parasite FV, at least in the FcB1 strain [22]. However, contrary to T5 that is highly specific for PfA-M1, BTA does also inhibit PfA-M17 (Ki 4000 nM versus 260 nM on PfA-M1). The data obtained on this strain, using the highly selective T5 inhibitor for PfA-M1 over PfA-M17, suggest that PfA-M1 could have additional functional roles besides the one involved in the haemoglobin degradation, that deserves further functional investigations.

In conclusion, this is the first report denoting in vitro and in vivo inhibitory activity for a new compound targeting PfA-M1 but not PfA-M17, further strengthening the potential of this target for anti-malarial research.

## Additional file

**Additional file 1.** *In vitro* and in vivo profile of compound T5. Description of data: The data, detailed in this additional file, comprises a summary table for T5 pharmacokinetic properties, followed by descriptions of the various corresponding assays.

## Abbreviations

Ala-pNA: alanine-para-nitroanilide
D.p.i.: day post-infection
FV: food vacuole
iRBC: infected red blood cells
PbA-M1: *Plasmodium berghei* M1 aminopeptidase
PcA-M1: *Plasmodium c. chabaudi* M1 aminopeptidase
PfA-M1: *Plasmodium falciparum* M1 aminopeptidase
PyA-M1: *Plasmodium yoelii* M1 aminopeptidase
