# Supplementary material for: Selective inhibition of PfA-M1, over PfA-M17, by an amino-benzosuberone derivative blocks malaria parasites development in vitro and in vivo
Source: Malar J. 2017 Sep 21;16:382. doi: 10.1186/s12936-017-2032-4 (PMC5609037; doi:10.1186/s12936-017-2032-4)
Supplement: Supplementary file 1 — Additional file 1. In vitro and in vivo profile of compound T5. Description of data: The data, detailed in this additional file, comprises a summary table for T5 pharmacokinetic properties, followed by descriptions of the various corresponding assays. [file 12936_2017_2032_MOESM1_ESM.pdf]

Additional file 1. *In vitro* and *in vivo* profile of compound T5.

The data, detailed below, comprises a summary table for T5 pharmacokinetic properties (Table S1), followed by descriptions of the various corresponding assays

Table S1. *In vitro* and *in vivo* profile of compound T5.

Compound	T5
rPfA-M1 K_i (µM)	0.05
rPfA-M17 K_i (µM)	> 100
Pf(3D7) IC_{50} (µM)[a]	11.2 ± 3.4 (n=5)
Pf(FcB1) IC_{50} (µM)	6.5 ± 2.4 (n=7)
Toxicity towards L6 cells, CC_{50} (µM)	141.0 ± 12.1 (n=4)
MWT (Da)	287.78
CHI	73
$logD_{7.4}$ calcd [b]	2.51
human microsomal stability, Clint (µL/min/mg protein)	18
kinetic solubility (µM)	151
human Plasma Protein Binding (% Bound)	83
PAMPA Permeability, Pe (x10^{-6} cm/s)	7.78
CYP inhibition IC_{50} (µM)	2C9, >10; 2D6, >10; 3A4, 5.7.
Intravenous PK Parameters(3mg/kg)	
Clb (mL/min/kg)	43
Vd_{ss} (L/kg)	7.4
terminal half-life, $T_{1/2}$ (min)	132
Oral PK Parameters (3mg/kg)	
C_{max} (µmol/L)	0.8
T_{max} (h)	0.25
Oral AUC_{0-8} (ng.min/mL)	26275
Bioavailability F(%)	41
Intraperitoneal PK Parameters (3mg/kg)	
C_{max} (µmol/L)	1.3
T_{max} (h)	0.25
Oral AUC_{0-8} (ng min/mL)	31521
Bioavailability F(%)	49
In vivo efficacy in Peter's test [c]	40% reduction in parasitemia (4 x 12 mg/kg, qd, IP)<br><br>44% reduction in parasitemia (4 x 24 mg/kg, qd, IP)

[a] Pf(3D7) is a chloroquine-susceptible strain of *P. falciparum*, whereas Pf(FcB1) is a chloroquine-resistant strain of *P. falciparum.* [b] calculated from CHI. [c] Pharmacokinetic and efficacy studies were carried out using compound T5 as the HCl salt.

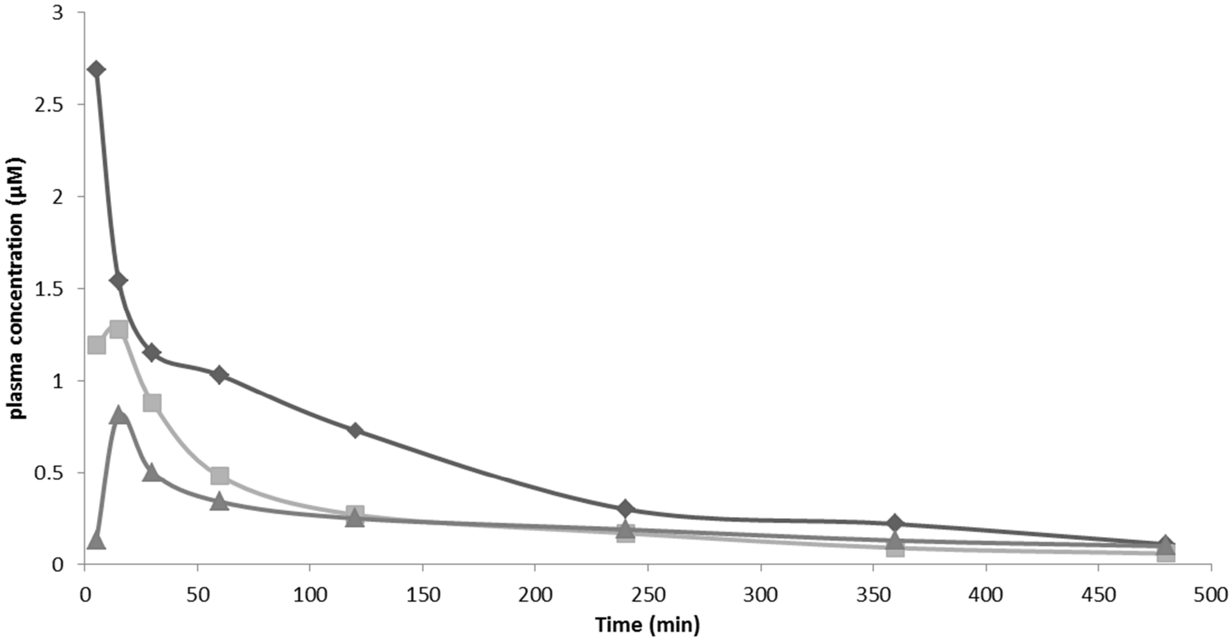

Figure S1. Concentration-time profile. Plasma concentration of T5 following intravenous (◆), intraperitoneal (■), oral (▲) administration of T5 (3mk/kg) to female CD-1 mice. Data are presented as the mean of n = 3 replicates.

Pharmacological properties

Determination of pharmacological properties of T5 was performed by TechMed^{ILL}, ESBS, Illkirch, France. Compounds are stored as 10 mM solution in DMSO at 4°C.

Solubility assays

Kinetic solubility was measured by diluting 20 µL of 10mM DMSO stock solution of compound T5 in a 980 µL pH 7.4 phosphate-buffered saline (PBS) with the following composition: 137.5 mM NaCl, 2.7 mM KCl, 4.3 mM Na_2HPO_4, and 1.4 mM KH_2PO_4. Samples were shaken during 16 hours at 20-22°C. After ultracentrifugation at 15000g for 10 min at 20°C, the concentration in the supernatant was measured by a HPLC procedure on a kinetex 2.6µm C18 100A 50x2.1 mm column using a calibration line established for the compound by diluting the 10 mM DMSO stock solution to adapted concentrations. The injection volume was 20 µL, the mobile phase flow rate was 2 mL/min and the following program was applied for the elution: 0-0.1 min, 5% B; 0.1-2.6 min, 5-95% B; 2.6-3.1 min, 95% B; 3.1-3.3 min, 95-5% B and 3.3-6 min, 5% B. Solvent A was a mixture of 0.05% trifluoroacetic acid in water and solvent B was acetonitrile. The detection wavelength was 254 nm and the retention time was 1.64 min.

Thermodynamic solubility was measured by dissolving 1mg of compound T5 in a 500 µL pH 7.4 phosphate-buffered saline (PBS) with the following composition: 137.5 mM NaCl, 2.7 mM KCl, 4.3 mM Na_2HPO_4, and 1.4 mM KH_2PO_4 supplemented or not with 10% Kolliphor EL, 15% Kolliphor HS15, 10% polysorbate 80, 50% PEG 400 or 10% hydroxypropyl-β-cyclodextrin. Samples were shaken during 24 hours at 20-22°C. Saturation was confirmed by the presence of undissolved powder. After ultracentrifugation at 15000g for 10 min, the concentration in the supernatant was measured by a HPLC procedure on a kinetex 2.6µm C18 100A 50x4.6 mm column using a calibration line established for the compound by diluting the 10 mM DMSO stock solution to adapted concentrations.

The injection volume was 20 µL, the mobile phase flow rate was 2 mL/min and the following program was applied for the elution: 0-0.1 min, 5% B; 0.1-2.6 min, 5-95% B; 2.6-3.1 min, 95% B; 3.1-3.3 min, 95-5% B and 3.3-6 min, 5% B. Solvent A was a mixture of 0.05% trifluoroacetic acid in water and solvent B was acetonitrile. The detection wavelength was 254 nm and the retention time was 1.64 min.

Table S2. Thermodynamic solubilities of compound T5 in PBS with different excipients.

**Thermodynamic solubility in PBS (pH 7.4)**	
excipient	(mM)
10% Kolliphor EL	0.046 ± 0.004
15% Kolliphor HS15	0.017± 0.002
10% polysorbate 80	0.325 ± 0.012
50% PEG 400	2.8 ± 0.1
10% hydroxypropyl-□-cyclodextrin	5.5 ± 0.2

CHI – logD determination

Chromatographic Hydrophobicity Indices (CHI) were determined according to an original procedure, based on a reverse phase fast HPLC gradient on a Luna C18(2) 5µ 100A 50 x 4.6mm column. First, a solution with 10 reference compounds with known CHI values was injected onto the HPLC to generate a calibration line from their retention time (CHI = 59.049*t_R – 56.021, R^2 = 0.9932). The concentration of the mixture was 0.1 mg/mL for each compound and the injected volume was 5 µL. T5 was analysed on the same system. The 10 mM DMSO stock solution was diluted to 200 µM in CH_3CN / 50 mM ammonium acetate pH 7.4 (3:7 v/v) and 5 µL were injected. The mobile phase flow rate was 2 mL/min and the following program was applied for the elution: 0-0.1 min, 0% B; 0.1-2.6 min, 0-100% B; 2.6-3.1 min, 100% B; 3.1-3.3 min, 100-0% B and 3.3-6 min, 0% B. Solvent A was 50 mM aqueous ammonium acetate pH 7.4 and solvent B was acetonitrile. The detection wavelength was 254 nm. The calibration line equation was used to determine the CHI values of T5. As CHI correlates closely with $LogD_{7.4}$ octanol/water partition coefficients, we estimated $LogD_{7.4}$ values for the compound. Estimation of logD is calculated using the correlation equation obtained from 14 known drugs by TechMedILL ($LogD_{7.4}$ = 0.0515*CHI - 1.2464, R^2 = 0.86).

Intrinsic Clearance (Cl_{int}) Determination

A 100 µM solution was firstly prepared by diluting 10 µL of 10 mM DMSO stock solution in 990 µL of a water/acetonitrile mixture (1:1 v/v). This solution was then diluted 1/100 in a 100 mM phosphate buffer (pH 7.4) containing human liver microsomes (0.5 mg/mL), 3 mM $MgCl_2$ and 1 mM NADPH. 400 µL of this solution was incubated at 37°C (initial compound concentration 1 µM). At time zero and then at 15, 30, 45 and 60 min, two aliquots (70 µL) of the incubation mixture were removed and diluted with acetonitrile (70 µL) to stop the reaction. Samples were stirred for 3 min, sonicated for 3 min and centrifuged at 15000g for 5 min at 4°C. The percentage of remaining compound was

determined by LC-MS/MS by measuring the area under the peak of the compound on the chromatogram using an UHPLC on a kinetex 2.6µm C18 100A 50x2.1 mm column coupled with a Shimadzu LCMS-8030 triple quadrupole.

1 µL was injected. The mobile phase flow rate was 0.5 mL/min and the following program was applied for the elution: 0 min, 5% B; 0-1.2 min, 5-95% B; 1.2-1.4 min, 95% B; 1.4-1.42 min, 95-5% B and 1.42-2.8 min, 5% B. Solvent A was a mixture of 0.05% formic acid in water and solvent B was acetonitrile. The detection wavelength was 254 nm. The concentration of parent compound versus time was subsequently modelled to an exponential decay function to determine the first order rate constant for parent compound depletion (k), which was ultimately used in the estimation of microsomal clearance. Negative control was performed by replacing NADPH by a similar volume of buffer. Positive control was performed using testosterone (experimental $T_{1/2}$ 27 min, literature value 32 min).

PAMPA assay

A 1 mM working solution was prepared by diluting 50 µL of 10 mM DMSO stock solution in 450 µL of DMSO. Donor solution (50 µM compound in 5% v/v DMSO/PBS, 1% Lucifer yellow) was prepared by diluting 1/20 the working solution in 1% v/v Lucifer yellow/PBS. An aliquot (150 µL) of the donor solution was added to the donor plate well which contained a PVDF filter membrane (area 0.24 cm^2) precoated with 5 µL of 2% phosphatidylcholin in dodecane. The PTFE acceptor plate well was filled with 300 µL of PBS, and the donor plate and acceptor plate were combined and incubated for 16 h at 20°C. The plates were removed from the shaker, and the acceptor sample was prepared by mixing 270 µL of solution from the acceptor well with 130 µL of acetonitrile. The donor sample was prepared by diluting an aliquot of solution from the donor well (20 µL) with PBS (250 µL) and acetonitrile (130 µL). The T0 sample was prepared by diluting an aliquot of donor solution (20 µL) with PBS (250 µL) and acetonitrile containing internal standard (130 µL). To determine the compound concentration at equilibrium, 150 µL of donor solution (50 µM compound in 5% v/v DMSO/PBS, 1% Lucifer yellow) were diluted with 300 µL PBS (pH 7.4). The prepared samples were analysed by LC-MS/MS by measuring the area under the peak (AUC) of the compound on the chromatogram using an UHPLC on a kinetex 2.6µm C18 100A 50x2.1 mm column coupled with a Shimadzu LCMS-8030 triple quadrupole. 1 µL was injected. The mobile phase flow rate was 0.5 mL/min and the following program was applied for the elution: 0 min, 5% B; 0-1.2 min, 5-95% B; 1.2-1.4 min, 95% B; 1.4-1.42 min, 95-5% B and 1.42-2.8 min, 5% B. Solvent A was a mixture of 0.05% formic acid in water and solvent B was acetonitrile. The detection wavelength was 254 nm.

The effective permeability value (Pe) was calculated using following equation,

$$Pe = -C\ Ln\left[1 - \frac{AUC_A}{AUC_{eq}}\right]$$

$$C = \frac{V_D \times V_A}{(V_D + V_A) \times area \times time}$$

Similar experiments were performed in parallel with chloramphenicol and testosterone used as negative (experimental log Pe = -6.4, literature value -7.0) and positive (experimental log Pe = -4.6, literature value -4.4) permeability markers, respectively.

Plasma protein binding protocol- Rapid Equilibrium Dialysis (RED) Assay Conditions

Single use RED device inserts were positioned in the PTFE RED base plate. Each insert consisted of 2 chambers separated by a vertically aligned semipermeable cellulose membrane with a molecular weight cut off (MWCO) of 12 kDa. Spiking solutions of T5 and warfarin were diluted in DMSO to 100 µM (10 µL of stock solution + 990 µL of DMSO).Human plasma was spiked with compound T5 or verapamil, which served as binding positive control, to achieve final concentrations of 1 µM. The final percentage of DMSO in the plasma incubation samples was 1%. Spiked plasma (200 µL) was added to the red chamber of the RED device insert and PBS (350 µL) was added to the white chamber. The samples were allowed to dialyze for 4 hours at 37°C in an orbitaler while agitating at a rate of 250 rpm. Each sample was incubated in duplicate. At the end of the incubation, 70 µL aliquots were removed from each chamber and matrix-matched, i.e., an equal volume of PBS was added to the plasma test incubation sample and an equal volume of plasma was added to the PBS test incubation sample. Samples where protein precipitated with 350 µL acetonitrile were freezed before analysis. After thawing, the samples were vortex-mixed for 3 min and sonicated for 3 min. The samples were then centrifuged at 15 000g for 5 min at 4°C to precipitate any proteins. The supernatants were analysed by UHPLC on a kinetex 2.6µm C18 100A 50x2.1 mm column coupled with a Shimadzu LCMS-8030 triple quadrupole using a calibration line established for the compound by diluting a 10 mM DMSO stock solution to adapted concentrations (0.05-2 µM). 1 µL of each sample was injected. The mobile phase flow rate was 0.5 mL/min and the following program was applied for the elution: 0 min, 5% B; 0-1.2 min, 5-95% B; 1.2-1.4 min, 95% B; 1.4-1.42 min, 95-5% B and 1.42-2.8 min, 5% B. Solvent A was a mixture of 0.05% formic acid in water and solvent B was acetonitrile. The detection wavelength was 254 nm. The unbound fraction was determined as the ratio of the peak area in buffer to that in plasma. Positive control was performed using verapamil (experimental F_u 10%, literature value 8%).

CYP inhibition

The compound T5 (0.1−10 µM) was incubated with human liver microsomes (BD Biosciences, Switzerland) (0.05, 0.16 and 0.2 mg/mL, for 3A4, 2D6 and 2C9 inhibition assays, respectively) and NADPH (1 mM) in a 100 mM potassium phosphate buffer pH 7.4 in the presence of a cytochrome P450 isoform-specific probe substrate (diclofenac for CYP2C9, bufuralol hydrochloride for CYP2D6, and midazolam maleate for CYP3A4) in a 37 °C water bath for the appropriate incubation time (10, 30 and 15 min for 3A4, 2D6 and 2C9, respectively). The reactions were terminated by adding ice-cold acetonitrile containing an internal standard followed by vortex mixing. The samples were then centrifuged at 15 000g for 10 min at 4 °C to precipitate the microsomal proteins. After centrifugation, the supernatants were analyzed by an UHPLC on a kinetex 2.6µm C18 100A 50x2.1 mm column coupled with a Shimadzu LCMS-8030 triple quadrupole. For each isoform, a selective inhibitor (sulfaphenazole for CYP2C9, quinidine for CYP2D6, and ketoconazole for CYP3A4) was used as a positive control. The IC50 values were calculated by nonlinear regression analysis from the plotted remaining metabolic activity at each test compound concentration.

In vivo *Pharmacokinetics*

Female CD-1 mice were intravenously administered a 2 mL/kg solution of T5 (3mg/kg dose; 1.5mg/mL of T5 dissolved in PBS solution containing 10% w/v hydroxypropyl-β-cyclodextrin). In

additional studies, mice were intraperitoneally administered a 10 mL/kg solution of T5 (3mg/kg dose; IV solution diluted 1:5 with PBS solution) or orally administered a 10 mL/kg solution of T5 (3mg/kg dose; IV solution diluted 1:5 with distilled water). The animals were sacrificed at 5, 15 and 30 minutes, 1, 2, 4, 6, and 8 hours after administration (n=3 per time), and blood samples were collected by cardiac puncture. The plasma was separated by centrifugation and stored frozen at -80°C. 400 µL of each plasma sample were mixed with 1 mL of acetonitrile, followed by vortex mixing for 3 min and sonication for 3 min. The samples were then centrifuged at 15 000g for 5 min at 4°C to precipitate any proteins. After centrifugation, the concentration in the supernatants were analyzed by UHPLC on a kinetex 2.6µm C18 100A 50x2.1 mm column coupled with a Shimadzu LCMS-8030 triple quadrupole using a calibration line established for the compound by diluting a 10 mM DMSO stock solution to adapted concentrations (0.05-2 µM). 1 µL was injected. The mobile phase flow rate was 0.5 mL/min and the following program was applied for the elution: 0 min, 5% B; 0-1.2 min, 5-95% B; 1.2-1.4 min, 95% B; 1.4-1.42 min, 95-5% B and 1.42-2.8 min, 5% B. Solvent A was a mixture of 0.05% formic acid in water and solvent B was acetonitrile. The detection wavelength was 254 nm. Pharmacokinetic parameters were derived from the blood concentration time curve using the non-compartmental analysis of PK Solver 2.0 [1].

1. Zhang Y, Huo M, Zhou J, Xie S: **PKSolver: An add-in program for pharmacokinetic and pharmacodynamic data analysis in Microsoft Excel.** *Comput Methods Programs Biomed* 2010, **99:**306-314.
